# Conventional MRI features can predict the molecular subtype of adult grade 2–3 intracranial diffuse gliomas

**DOI:** 10.1007/s00234-022-02975-0

**Published:** 2022-05-24

**Authors:** Arian Lasocki, Michael E. Buckland, Katharine J. Drummond, Heng Wei, Jing Xie, Michael Christie, Andrew Neal, Frank Gaillard

**Affiliations:** 1 Department of Cancer Imaging, Peter MacCallum Cancer Centre, Melbourne, VIC Australia; 2 Sir Peter MacCallum Department of Oncology, The University of Melbourne, Parkville, VIC Australia; 3 Department of Neuropathology, Royal Prince Alfred Hospital, Camperdown, NSW Australia; 4 School of Medical Sciences, University of Sydney, Camperdown, NSW Australia; 5 Department of Neurosurgery, The Royal Melbourne Hospital, Parkville, VIC Australia; 6 Department of Surgery, The University of Melbourne, Parkville, VIC Australia; 7 Centre for Biostatistics and Clinical Trials, Peter MacCallum Cancer Centre, Melbourne, VIC Australia; 8 Department of Anatomical Pathology, The Royal Melbourne Hospital, Parkville, VIC Australia; 9 Department of Neurology, The Royal Melbourne Hospital, Parkville, VIC Australia; 10 Department of Neuroscience, Central Clinical School, Monash University, Clayton, VIC Australia; 11 Department of Radiology, The Royal Melbourne Hospital, Parkville, VIC Australia; 12 Department of Radiology, The University of Melbourne, Parkville, VIC Australia

**Keywords:** Glioma, Glioblastoma, MRI, Radiogenomics, T2-FLAIR mismatch

## Abstract

**Purpose:**

Molecular biomarkers are important for classifying intracranial gliomas, prompting research into correlating imaging with genotype (“radiogenomics”). A limitation of the existing radiogenomics literature is the paucity of studies specifically characterizing grade 2–3 gliomas into the three key molecular subtypes. Our study investigated the accuracy of multiple different conventional MRI features for genotype prediction.

**Methods:**

Grade 2–3 gliomas diagnosed between 2007 and 2013 were identified. Two neuroradiologists independently assessed nine conventional MRI features. Features with better inter-observer agreement (κ ≥ 0.6) proceeded to consensus assessment. MRI features were correlated with genotype, classified as IDH-mutant and 1p/19q-codeleted (IDH^mut^/1p19q^codel^), IDH-mutant and 1p/19q-intact (IDH^mut^/1p19q^int^), or IDH-wildtype (IDH^wt^). For IDH^wt^ tumors, additional molecular markers of glioblastoma were noted.

**Results:**

One hundred nineteen patients were included. T2-FLAIR mismatch (stratified as > 50%, 25–50%, or < 25%) was the most predictive feature across genotypes (p < 0.001). All 30 tumors with > 50% mismatch were IDH^mut^/1p19q^int^, and all seven with 25–50% mismatch. Well-defined margins correlated with IDH^mut^/1p19q^int^ status on univariate analysis (p < 0.001), but this related to correlation with T2-FLAIR mismatch; there was no longer an association when considering only tumors with < 25% mismatch (p = 0.386). Enhancement (p = 0.001), necrosis (p = 0.002), and hemorrhage (p = 0.027) correlated with IDH^wt^ status (especially “molecular glioblastoma”). Calcification correlated with IDH^mut^/1p19q^codel^ status (p = 0.003). A simple, step-wise algorithm incorporating these features, when present, correctly predicted genotype with a positive predictive value 91.8%.

**Conclusion:**

T2-FLAIR mismatch strongly predicts IDH^mut^/1p19q^int^ even with a lower threshold of ≥ 25% mismatch and outweighs other features. Secondary features include enhancement, necrosis and hemorrhage (predicting IDH^wt^, especially “molecular glioblastoma”), and calcification (predicting IDH^mut^/1p19q^codel^).

## Introduction

The addition of molecular biomarkers as a key component of glioma characterization in the 2016 update of the World Health Organization (WHO) Classification of Tumors of the Central Nervous System [1] (WHO 2016) has led to research into correlating imaging features with these molecular markers, known as radiogenomics or imaging genomics. Radiogenomics can aid clinical management in a variety of scenarios [2], and arguably, its potential value is increasing further as a greater number of molecular biomarkers enter clinical practice, based on the recommendations of cIMPACT-NOW [3] and the recent 2021 WHO classification [4] (WHO 2021). Two molecular markers have provided the basis of classification of lower grade (WHO grades 2–3) diffuse gliomas (LGG) since WHO 2016: isocitrate dehydrogenase-1 or -2 variant status (IDH; either IDH-mutant or IDH-wildtype) and 1p/19q-codeletion (combined loss of both the short arm of chromosome 1 and the long arm of chromosome 19) [1]. These two markers allow classification of LGG into one of three subtypes: IDH-mutant with 1p/19q-codeletion (IDH^mut^/1p19q^codel^), IDH-mutant with intact 1p/19q (IDH^mut^/1p19q^int^), and IDH-wildtype (IDH^wt^) [1]. As such, these markers have been the focus of substantial research. A notable addition in WHO 2021 has been the addition of molecular criteria to the diagnosis of glioblastoma (“molecular GBM”), which allows a phenotypic grade 2–3 IDH^wt^ glioma to be diagnosed as a glioblastoma if at least one of three additional molecular features is demonstrated [4]. If these molecular features are absent, the tumor is labeled IDH^wt^ NEC (not elsewhere classified) [4]. Given the recency of this classification, however, there is a paucity of radiogenomic research examining these additional molecular markers.

Radiogenomics research on routine clinical MRI scans can be broadly split into conventional visual assessment by radiologists (conventional radiogenomics) and analysis utilizing artificial intelligence (AI) techniques (AI radiogenomics). As would be expected, there has been a recent shift towards research into AI techniques, but these have not yet reached clinical practice and there remain some limitations [5]. In contrast, predicting genotype by conventional imaging assessment can be immediately incorporated into routine clinical practice [2]. In addition, results of conventional radiogenomics research provide a basis for correlations identified by AI. For example, a study utilizing deep learning and multiparametric imaging found that the addition of CT improved the prediction obtained by using only MRI and positron emission tomography [6], attributed to the correlation between calcifications and 1p/19q-codeletion [7, 8]. Similarly, conventional radiogenomics can suggest which sequences and techniques are likely to be most helpful for incorporation into AI radiogenomics research.

A recent systematic review found that the T2-FLAIR mismatch sign was the most distinctive conventional radiogenomic feature of LGG [5]. High specificity, approaching 100%, has been demonstrated in several cohorts, with good inter-rater agreement [7, 9–12]. An acknowledged limitation of the T2-FLAIR mismatch sign, however, is its limited sensitivity, being less than 50% in a recent pooled meta-analysis [12]. A variety of other conventional MRI features have been investigated, including tumor location, tumor margin appearances, internal signal characteristics, enhancement, cortical involvement, and magnetic susceptibility (including calcifications and hemorrhage), with varying results [5]. In addition, some features have been investigated in the context of glioblastoma, in particular assessing the morphology of noncontrast-enhancing tumor as a predictor of IDH status [7, 13], but these have not yet been investigated in the context of LGG to our knowledge.

The above systematic review also identified several limitations of the existing literature [5]. For example, only a minority of studies have reported inter-observer comparisons, and most studies investigated either IDH or 1p/19q status, or both independently, with classification into the three molecular subtypes according to the 2016 WHO criteria being less common [5]. A further limitation of the existing literature is that it is unclear how best to incorporate and weight multiple imaging features into a conventional radiogenomic prediction that can be applied in everyday clinical practice. Algorithmic approaches are less common in the literature and have generally not allowed for a prediction of all three molecular subtypes [7, 9, 14, 15].

The purpose of this study was to investigate the accuracy of multiple different conventional MRI features for the prediction of the three molecular subtypes of LGG, as well as distinguishing between molecular GBM and IDH^wt^ NEC, with the goal of developing a user-friendly and robust algorithm for the prediction of LGG molecular subtype based on conventional MRI features.

## Methods

### Patient identification

Institutional Human Research Ethics Committee approval was obtained (HREC number QA2017093). Patients with a new diagnosis of a grade 2–3 intracranial glioma between September 2007 and December 2013 (to allow for long term survival data) were identified through the Central Nervous System Tumor Database at our hospital and previous research at our institution [7, 16]. All were adult patients.

### Imaging assessment

Only patients with at least T2WI, FLAIR, and pre- and post-contrast T1WI were included. MRIs had been performed on a variety of scanners, including at external institutions. Relevant CT images were also reviewed to assess for calcification, preferentially pre-operative, or otherwise post-operative where relevant (and accounting for interval resection). Susceptibility-sensitive sequences were also reviewed, including phase images where available.

### Imaging features

**Fig. 1:**
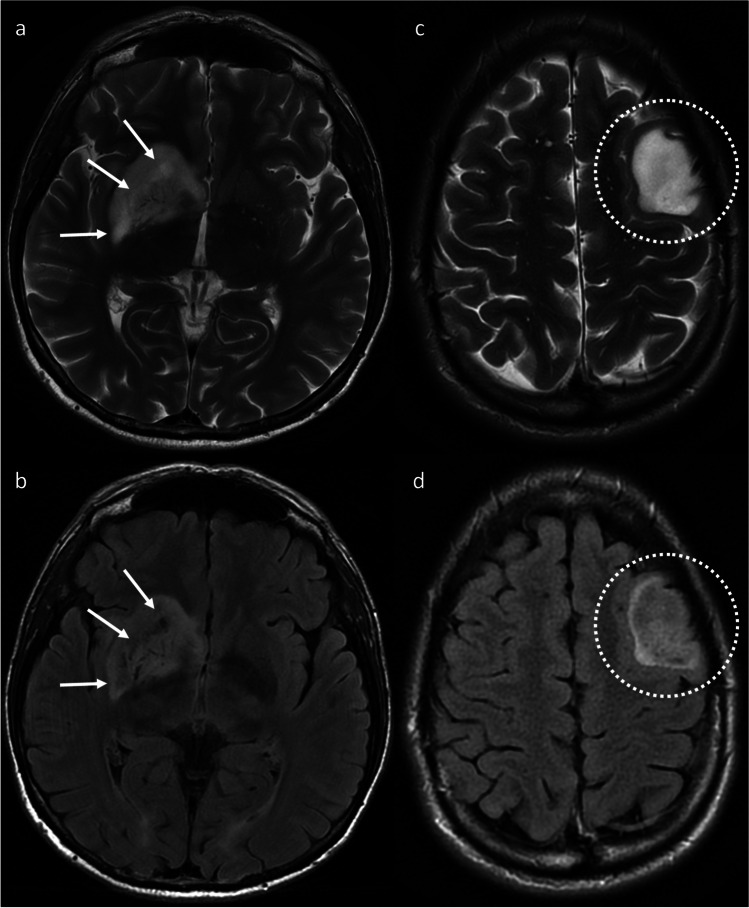
Axial T2WI (top row) and FLAIR (bottom row) images of two patients with IDH^mut^/1p19q^int^ LGG. The first patient (**a**, **b**) has a tumor centered on the right lentiform nucleus, demonstrating 25–50% T2-FLAIR mismatch. The arrows highlight areas with high T2 signal and substantially lower signal on FLAIR. The second patient (**c**, **d**) has a left frontal lobe tumor with > 50% T2-FLAIR mismatch and well-defined tumor margins

**Fig. 2:**
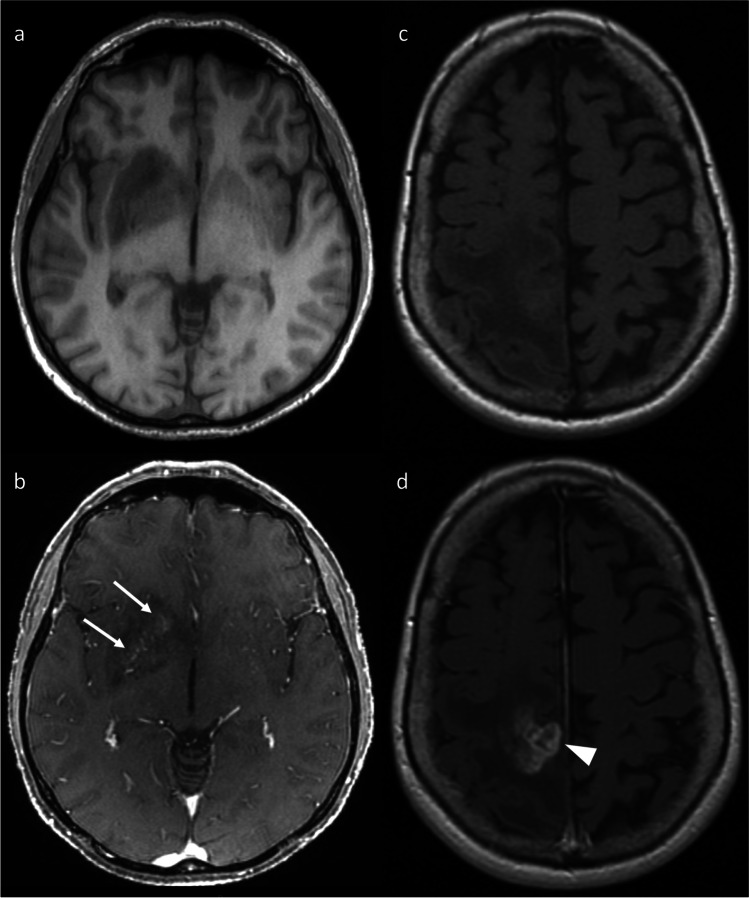
Axial pre-contrast T1WI (top row) and post-contrast T1WI (bottom row) demonstrating different patterns of enhancement. The first patient (**a**, **b**) has an IDH^mut^/1p19q^int^ LGG demonstrating wispy enhancement, shown by the arrows. The second patient (**c** & **d**) has a right parietal lobe tumor with nodular enhancement, evidence of necrosis (arrowhead), and ill-defined tumor margins

The following imaging features were assessed for all patients, based on prior published definitions where possible:

- Tumor location, recorded as either frontal (all/predominantly frontal, but not insular/temporal), temporal (all/predominantly temporal, but not insular/frontal); fronto-temporal (+ / − insular) or none of the above (no/minimal frontal and/or temporal lobe involvement)
- T2-FLAIR mismatch [11], stratified as either > 50% of the tumor volume (distinguished from edema [13]), 25–50% or < 25% (Fig. 1)
- Well-defined tumor margins, defined as > 75% of the tumor circumference considered geographically marginated [17]
- Homogeneous (on both T1WI and T2WI)
- Magnetic susceptibility, recorded as either none, consistent with calcification, convincingly representing hemorrhage or indeterminate between calcification and hemorrhage
- Enhancement, stratified as either none, wispy (subtle and ill-defined, without discrete nodularity) and distinct (Fig. 2)
- Presence of central necrosis (requiring a central non-enhancing area surrounded by a complete enhancing ring)
- The presence of eccentric FLAIR hyperintensity consistent with infiltrative noncontrast-enhancing tumor (nCET) [13]
- The presence of a mass-like morphology of nCET [7]
- Predominant cortical involvement of nCET

Assessment was performed independently by two neuroradiologists from different institutions with subspecialty expertise in neuro-oncology (having 8 and 13 years of experience), blinded to molecular status. After this read, inter-rater agreement was calculated for all features and directed the features to be assessed through consensus review, based on inter-observer agreement yielding κ ≥ 0.6. Features with lower inter-rater agreement (κ < 0.6) were not assessed further.

### Histological assessment

Histological diagnoses were obtained from the database and confirmed with the formal pathology report (in particular, confirming the diagnosis of a grade 2–3 diffuse glioma). Where available, IDH1-R132H immunohistochemistry and 1p/19q-codeletion results (the latter typically performed by fluorescence in situ hybridization) were obtained from hospital pathology results (performed as part of routine patient care) and previous research performed at our institution [7, 16]. IDH pyrosequencing results were also available for a small number of patients through prior research [18]. Complete molecular characterization required *IDH1* codon 132 and *IDH2* codon 172 sequencing if IDH1-R132H immunohistochemistry was negative, and determination of 1p/19q-codeletion status for patients with IDH mutation detected by any method. For patients without complete molecular characterization based on existing data, next-generation sequencing (NGS) was performed to determine IDH and 1p/19q-codeletion status, utilizing previously reported methodology [19]. For patients undergoing NGS, the output also included *TERT* promoter variant status, + 7/ − 10 and *EGFR* copy number status according to the updated criteria for glioblastoma in WHO 2021 [4].

Based on the above results, all tumors were classified as either IDH^mut^/1p19q^codel^, IDH^mut^/1p19q^int^, or IDH^wt^. Patients in whom definitive molecular characterization as one of these three genotypes was not possible—most commonly due to insufficient or inadequate material being available for NGS—were excluded. In addition, IDH^wt^ tumors were further divided into molecular GBM and IDH^wt^ NEC [4].

### Statistical analysis

All statistical analyses were performed in R (R version 4.0.3 (2020–10-10)) using standard and validated statistical procedures. Descriptive statistics of baseline characteristics of all included patients were reported. Continuous variables were described as median, minimum, and maximum, and categorical variables were described as counts and percentages. Cohen’s κ-coefficient (κ) was used to measure the degree of agreement between a pair of variables. Logistic regression analysis was used to assess for associations between MRI features and molecular subtype. Odds ratios (OR) with 95% confidence intervals (CI) and p-values were calculated. Multivariable logistic regression analysis included all important risk factors with p < 0.1. A p value less than 0.05 was defined as statistically significant.

## Results

### Patient results

Twenty-three patients were excluded due to incomplete molecular characterization. A further three patients were excluded due to the identification of H3 histone mutations (two K27M, one G34R). Thus, the study population of 119 patients included 58 (48.7%) IDH^mut^/1p19q^int^ tumors, 32 (26.9%) IDH^wt^ and 29 (24.4%) IDH^mut^/1p19q^codel^. The patients had a median age of 38 years (range 17–83 years), and 62% were male. NGS was performed on the majority of patients (108 of 119), with only 11 tumors already adequately characterized molecularly based on existing data. Only one patient had had IDH^wt^ status confirmed through IDH pyrosequencing; the remaining 31 IDH^wt^ tumors underwent NGS. Twenty-four (77.4%) of these met the criteria for molecular GBM [4].

After single-reader assessment, the following features had at least moderate inter-observer agreement (κ ≥ 0.6) and proceeded to the consensus read: location (*κ* = 0.939), necrosis (*κ* = 0.766), well-defined tumor margins (*κ* = 0.723), enhancement (*κ* = 0.706), magnetic susceptibility (*κ* = 0.622), and T2-FLAIR mismatch (*κ* = 0.616). The following features had a lower inter-observer agreement and were excluded from the subsequent assessment: eccentric nCET (*κ* = 0.51), homogeneous signal (*κ* = 0.481), cortical involvement (*κ* = 0.272), and mass-like nCET (*κ* = 0.231).

The frequency of each MRI feature based on tumor subtype is presented in Table 1. Concordant with the aforementioned literature [12], the T2-FLAIR mismatch sign was highly predictive of an IDH^mut^/1p19q^int^ tumor (p < 0.001), including with a lower threshold of T2-FLAIR mismatch, and this was the single most predictive feature across molecular subtypes. All 30 tumors with > 50% mismatch were IDH^mut^/1p19q^int^, as well as all seven with 25–50% mismatch (p < 0.001). Combining both groups, all 37 tumors with ≥ 25% mismatch were IDH^mut^/1p19q^int^, producing a specificity of 100% and sensitivity of 63%. Eight (21.6%) of the 37 tumors with ≥ 25% mismatch demonstrated solid enhancement (all in the > 50% group), with a further eight demonstrating wispy enhancement. The presence of ≥ 25% mismatch predicted IDH^mut^/1p19q^int^ status in both enhancing and non-enhancing tumors.

**Table 1:** The frequency of each MRI feature based on tumor molecular subtype

MRI feature	Tumor subtype			
	IDH^m^/1p19q^int^	IDH^m^/1p19q^codel^	IDH^wt^	Total
	*n* = 58	*n* = 29	*n* = 32	*n* = 119
Tumor location				
Frontal	27 (46.6%)	16 (55.2%)	9 (28%)	52 (43.7%)
Fronto-temporal	8 (13.8%)	4 (13.8%)	3 (9%)	15 (12.6%)
Temporal	12 (20.7%)	2 (6.9%)	5 (16%)	19 (16.0%)
None of the above	11 (19.0%)	7 (24.1%)	15 (47%)	33 (27.7%)
T2-FLAIR mismatch				
> 50%	30 (51.7%)	0	0	30 (25.2%)
25–50%	7 (12.1%)	0	0	7 (5.9%)
< 25%	21 (36.2%)	29 (100.0%)	32 (100%)	82 (68.9%)
Well-defined				
Yes	29 (50.0%)	5 (17.2%)	2 (6%)	36 (30.3%)
No	29 (50.0%)	24 (82.8%)	30 (94%)	83 (69.7%)
Susceptibility				
Calcifications	1 (1.7%)	7 (24.1%)	2 (6%)	10 (8.4%)
Hemorrhage	1 (1.7%)	0	4 (12%)	5 (4.2%)
Nonspecific	7 (12.1%)	2 (6.2%)	4 (12%)	13 (10.9%)
None	49 (84.5%)	20 (69.0%)	22 (69%)	91 (76.5%)
Enhancement				
Yes	14 (24.1%)	3 (10.3%)	19 (59%)	36 (30.3%)
Wispy	13 (22.4%)	7 (24.1%)	2 (6%)	22 (18.5%)
No	31 (53.4%)	19 (65.5%)	11 (34%)	61 (51.3%)
Necrosis				
Yes	6 (10.3%)	2 (6.9%)	11 (34%)	19 (16.0%)
No	52 (89.7%)	27 (93.1%)	21 (66%)	100 (84.0%)

On univariate analysis, well-defined tumor margins correlated with IDH^mut^/1p19q^int^ status (OR 7.7, 95% CI 3.0–19.8, p < 0.001), with 29 (50.0%) of 58 IDH^mut^/1p19q^int^ tumors having well-defined margins. Most IDH^wt^ tumors (30 of 32, 93.8%, p = 0.003) had ill-defined margins, as well as 24 (82.8%) of 29 IDH^mut^/1p19q^codel^ tumors, limiting the distinction between these two subtypes. Importantly, well-defined tumor margins strongly correlated with T2-FLAIR mismatch (p < 0.001; contingency coefficient, *C* = 0.72), especially with > 50% mismatch (23 of 30 were well-defined, 76.7%). There was less correlation with tumors exhibiting 25–50% mismatch (two of seven with well-defined margins, 28.6%). Given the above association, well-defined margins no longer correlated significantly with IDH^mut^/1p19q^int^ status when considering only tumors with < 25% mismatch, with only four (19.0%) of the 21 IDH^mut^/1p19q^int^ tumors with < 25% mismatch exhibiting well-defined margins (p = 0.386). IDH^mut^/1p19q^int^ tumors were most commonly frontal (46.6%), IDH^mut^/1p19q^codel^ tumors were usually frontal (55.2%) and uncommonly temporal (6.9%), and IDH^wt^ tumors were most commonly neither frontal nor temporal (46.9%); however, there was no statistically significant correlation between tumor location and genotype.

The predictive value of magnetic susceptibility was limited, as the majority of tumors (91 of 119, 76.5%) demonstrated no susceptibility, and for a further 13 (10.9%), the distinction between calcification and hemorrhage was unclear. Ten tumors exhibited convincing calcification, and seven of these (70%) were IDH^mut^/1p19q^codel^ (OR 8.3, 95% CI 2.0–35.0, p = 0.004). However, most (22 of 29) IDH^mut^/1p19q^codel^ tumors did not have convincing calcification. There were no other particular features of the calcified, non-codeleted tumors that would have confidently suggested an alternate subtype: no tumors demonstrated calcification and ≥ 25% mismatch, and the one tumor with both calcification and necrosis was IDH^mut^/1p19q^codel^. Five tumors showed evidence of hemorrhage, of which four (80%) were IDH^wt^. Notably, an IDH^mut^/1p19q^int^ tumor with hemorrhage had ≥ 25% mismatch, so all four tumors with evidence of hemorrhage and < 25% mismatch were IDH^wt^. As for calcification, however, most (28 of 32) IDH^wt^ tumors did not demonstrate hemorrhage.

Enhancement and necrosis both correlated strongly with IDH^wt^ status (OR 5.1, 95% CI 2.0–12.8, p = 0.001 for enhancement; OR 5.2, 95% 1.8–14.5, p = 0.002 for necrosis), with 19 (52.8%) of 36 enhancing tumors and 11 (57.9%) of 19 necrotic tumors being IDH^wt^. Of the 32 IDH^wt^ tumors, 59.4% demonstrated enhancement and 34.4% had necrosis. Of note, necrosis was visualized in five tumors demonstrating ≥ 25% mismatch (all having > 50% mismatch), and all were IDH^mut^/1p19q^int^. Once tumors with ≥ 25% mismatch were excluded from the subgroup analysis, 78.6% of tumors demonstrating necrosis were IDH^wt^ (of which three also demonstrated evidence of hemorrhage). Enhancement and necrosis were least common in IDH^mut^/1p19q^codel^ tumors, with only three (10.3%) and two (6.9%) of the 29 IDH^mut^/1p19q^codel^ tumors exhibiting these features, respectively. Of note, tumors with solid enhancement were significantly less likely to be IDH^mut^/1p19q^codel^ (OR 0.2, 95% CI 0.1–0.7, p = 0.016). Enhancement and necrosis were both more common in molecular GBM (enhancement in 16/24, 80.0%; necrosis in 9/24, 37.5%) than IDH^wt^ NEC (enhancement in 2/7, 28.6%; necrosis in 1/7, 14.3%), and both differences were statistically significant (p < 0.001 and p < 0.001, respectively). Additionally, all four IDH^wt^ tumors with evidence of hemorrhage were molecular GBMs, while both IDH^wt^ tumors with calcification were IDH^wt^ NEC.

In terms of an overall algorithmic approach, identification of ≥ 25% mismatch was shown to be the key first step, allowing high confidence of IDH^mut^/1p19q^int^ status regardless of other features which might otherwise predict a different molecular subtype (such as necrosis). Once mismatch had been considered, the ability of other features (in particular well-defined tumor margins) to predict IDH^mut^/1p19q^int^ status diminished, but the ability to predict an IDH^wt^ tumor improved. Specifically, after excluding tumors with ≥ 25% mismatch, all tumors with evidence of hemorrhage were IDH^wt^, the OR for enhancement improved from 5.1 to 5.6, and the OR for necrosis increased from 5.2 to 8.2. One possible approach would be to assess for magnetic susceptibility (calcification and hemorrhage) after T2-FLAIR mismatch and then for the presence of enhancement. In our cohort, this would yield an overall positive predictive value (PPV) of 87.3%, and 60% of tumors would have a positive finding. Alternatively, the enhancement could be replaced by necrosis, improving the positive predictive value to 91.8%, but decreasing the number of tumors with a positive finding to 51%.

## Discussion

Our findings reinforce the high-positive predictive value of the T2-FLAIR mismatch sign, but go well beyond just confirming the findings of previous studies. Firstly, we have stratified tumors by the percentage of T2-FLAIR mismatch, adding and specifically assessing tumors with a smaller proportion of mismatch (25–50%). While most tumors with T2-FLAIR mismatch demonstrate > 50% mismatch, smaller percentages of mismatch are not uncommon, and it is unclear from the existing literature how such tumors should be considered. In the original description by Patel et al., T2-FLAIR mismatch was defined as the “presence or absence of complete/near-complete hyperintense signal on T2WI, and relatively hypointense signal on FLAIR except for a hyperintense peripheral rim” [11]. This implies, without explicitly defining, substantially greater than 50% mismatch. One study predominantly used a threshold of > 50% mismatch, though also assessed for 33–50% mismatch, with only one further tumor identified [7]. Our findings suggest that the percentage of mismatch required to consider the feature positive can be dropped to 25% without a drop in PPV (remaining at 100%), but with improved sensitivity. This accounts for the relatively larger proportion of IDH^mut^/1p19q^int^ tumors demonstrating mismatch in our cohort compared to those reported previously. We have also investigated the accuracy of the T2-FLAIR mismatch sign in enhancing tumors, which is less clear in the literature. There have been suggestions that the PPV may be lower in enhancing tumors [20], but this was not the case in our cohort. Given that all other features were less accurate, we suggest that T2-FLAIR remains a very helpful feature in enhancing tumors, including those with evidence of necrosis.

A lower threshold for T2-FLAIR mismatch is in line with a more recently described feature, named “fluid attenuation in non-contrast-enhancing tumor,” which has been shown to predict IDH mutations in glioblastoma [21]. This feature bears substantial similarities to the T2-FLAIR mismatch sign in LGG and was defined as “any tumor volume with hyperintense signal on T2WI and corresponding hypointense signal on FLAIR” [21]. This definition implies that less than 50% fluid attenuation—and potentially even less than 25%—would nevertheless allow this feature to be considered present. This suggests that grade 2–4 gliomas could be assessed together using a threshold of ≥ 25% mismatch, though this has yet to be formally assessed to our knowledge. Such analysis is particularly relevant in the context of WHO 2021, given that the distinction between tumor grade has become less important, with molecular status playing a greater role [4].

**Fig. 3:**
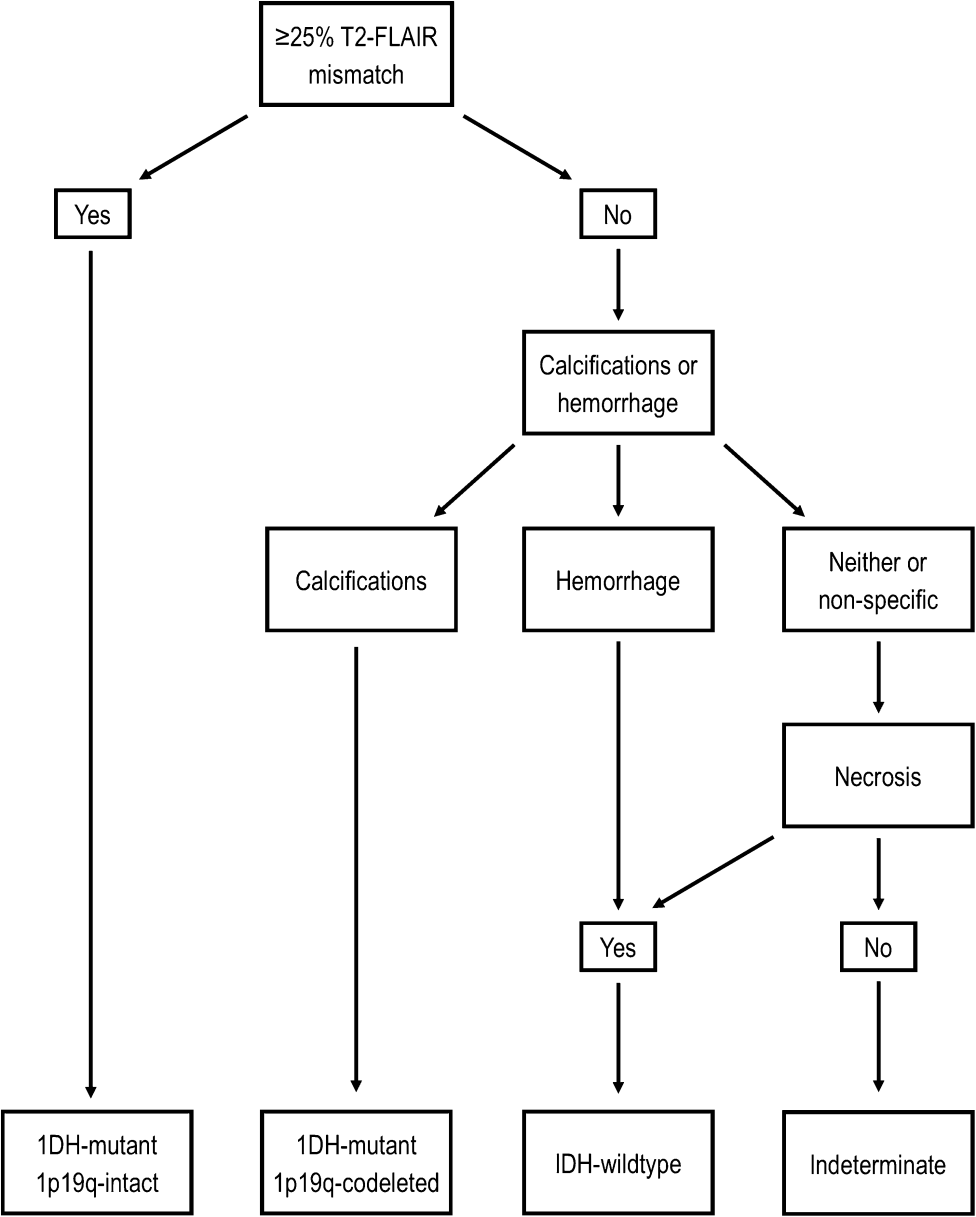
A proposed algorithm for the prediction of LGG genotype using visual features

Our data comparing the MRI features of IDH^wt^ tumors with and without additional molecular markers of GBM is another novel aspect of our work. We have shown that features predictive of IDH^wt^ status are predominantly present in molecular GBMs. We have also clarified some uncertainty regarding the overlap in MRI features, which has been under-studied in the literature. Specifically, while there have been some suggestions in the literature that well-defined margins predict an IDH^mut^/1p19q^int^ tumor [5], this seems to largely relate to an overlap with the T2-FLAIR mismatch sign; well-defined tumor margins have limited (and possibly no) predictive value once T2-FLAIR mismatch is accounted for. On the other hand, the value of other features, such as necrosis, improved once tumors with ≥ 25% mismatch were removed. This suggests that a step-wise, multi-feature algorithmic approach has value—a suggested algorithm is provided in Fig. 3. An alternate algorithm, considering necrosis ahead of magnetic susceptibility, achieved slightly lower accuracy in our cohort, as the one tumor with evidence of calcifications and necrosis was IDH^mut^/1p19q^codel^. A limitation of the assessment of magnetic susceptibility related to the uncertainty in distinguishing between calcifications and hemorrhage in many cases, due to the frequent unavailability of preoperative CT and/or SWI phase images in this dataset. The sensitivity, and potentially specificity, of the algorithm would be expected to increase if these were uniformly available.

ADC has shown promise for predicting genotype [5, 15], and the lack of inclusion of ADC measurements is a potential limitation of our study. Being a continuous variable, with overlap across the three genotypes, the ADC threshold can be adjusted to optimize either sensitivity or specificity, as is considered most appropriate [2]. We had not incorporated ADC as we had felt that it was unclear from the literature how best to do so, but now our findings provide some guidance. ADC would seem to have the greatest potential in the tumors without any of the key features described. However, ADC values are likely to correlate with T2-FLAIR mismatch to some extent—specifically, ADC values are likely to be high in tumor regions demonstrating T2-FLAIR mismatch. Thus, as with well-defined tumor margins, the predictive value may decrease once T2-FLAIR mismatch has been accounted for, and this warrants further investigation. Another use of ADC could be to supplement the secondary features (calcifications, hemorrhage, enhancement, and necrosis), by confirming that the ADC values lie within the expected range for the predicted genotype.

Despite including a larger number of features than many previous studies, a substantial proportion of tumors did not demonstrate any of the key features we identified. Thus, our goal of using conventional MRI features to predict all three molecular subtypes with high accuracy has not yet been realized. We had hoped that the inclusion of additional features (such as cortical involvement and the morphology of nCET) would allow some differentiation of these equivocal cases, but this was limited by the lower inter-rater agreement. There may nevertheless be some potential in these additional features, but a tighter definition is required. AI techniques which can visually represent the imaging appearances correlating with a particular glioma subtype, such as the technique of principal component analysis as reported by Chang et al. [22], hold promise in this regard. For example, we observed that there was variability in the appearances of tumors demonstrating cortical predominance, for example, in the degree of gray-white matter differentiation, but it was not clear how to objectively distinguish these patterns and whether they indeed reflected differences in the genotype. If AI techniques were able to identify subtle differences in the appearances of such tumors, this could facilitate a clearer definition for visual assessment and thus improve the inter-observer agreement, or at least supplement aspects of visual assessment which are more challenging.

Over-fitting is a common criticism of AI radiogenomics research and is also a potential consideration for the algorithmic approach we have suggested. Reassuringly, however, the individual features included in the final analysis have all been investigated previously, and our results are in line with previous reports. As such, we believe that similar results can be expected in a broader cohort, though subsequent validation will nevertheless be important. The retrospective nature of our study is a further limitation, as it is for much of the work in this field. We note that the patients in our cohort were from several years ago, and modern scanners could potentially achieve even better results. For example, the routine inclusion of susceptibility-weighted imaging including phase images should improve the detection of, and the distinction between, calcifications and hemorrhage, while modern volumetric post-contrast sequences will aid in the identification of enhancement and necrosis. Similarly, the inclusion of images from a variety of scanners, including external imaging, provides reassurance that the results can be translated to other institutions. We consider the proportions of our molecular subtypes to be concordant for the broader literature—for example, a cohort reported by The Cancer Genome Research Network had 50.0% IDH^mut^/1p19q^int^ tumors, 19.9% IDH^wt^, and 30.1% IDH^mut^/1p19q^codel^ [23]—though we note a slightly higher proportion of IDH^wt^ tumors and a slightly lower proportion of IDH^mut^/1p19q^codel^ tumors in our cohort compared to the above.

## Conclusions

The T2-FLAIR mismatch sign is highly predictive of IDH^mut^/1p19q^int^ status in LGG, including for tumors demonstrating post-contrast enhancement, necrosis, and smaller proportions of mismatch (≥ 25%). Well-defined tumor margins are associated with T2-FLAIR mismatch and are no longer independently predictive of IDH^mut^/1p19q^int^ status after accounting for T2-FLAIR mismatch. For tumors with < 25% mismatch, secondary predictive features include the presence of enhancement, necrosis, and/or hemorrhage (favoring an IDH^wt^ tumor, especially those with additional molecular features of glioblastoma) and the presence of calcifications (predicting IDH^mut^/1p19q^codel^).

## Data Availability

N/A.

## Abbreviations

AI: Artificial intelligence
GBM: Glioblastoma
IDH: Isocitrate dehydrogenase
IDH^mut^/1p19q^codel^: IDH-mutant with 1p/19q-codeletion
IDH^mut^/1p19q^int^: IDH-mutant with intact 1p/19q
IDH^wt^: IDH-wildtype
IDH^wt^ NEC: IDH-wildtype not elsewhere classified
LGG: Lower grade glioma (WHO grade 2–3)
nCET: Noncontrast-enhancing tumor
NGS: Next-generation sequencing
OR: Odds ratio
CI: Confidence interval
PPV: Positive predictive value
WHO: World Health Organization
